# Protein Phosphatase 4 Promotes Hepatocyte Lipoapoptosis by Regulating RAC1/MLK3/JNK Pathway

**DOI:** 10.1155/2021/5550498

**Published:** 2021-06-15

**Authors:** Xiuqing Huang, Guang Yang, Li Zhao, Huiping Yuan, Hao Chen, Tao Shen, Weiqing Tang, Yong Man, Jiarui Ma, Yanyan Ma, Lin Dou, Jian Li

**Affiliations:** ^1^The Key Laboratory of Geriatrics, Beijing Institute of Geriatrics, Beijing Hospital, National Center of Gerontology, National Health Commission; Institute of Geriatric Medicine, Chinese Academy of Medical Sciences, Beijing 100730, China; ^2^Department of Gastroenterology, Beijing Hospital, National Center of Gerontology, Institute of Geriatric Medicine, Chinese Academy of Medical Sciences, Beijing 100730, China; ^3^Department of Scientific Research, Qinghai University Affiliated Hospital, Xining 810001, China

## Abstract

Lipotoxicity-induced apoptosis, also referred to as lipoapoptosis, is one of the important initial factors promoting the progression from hepatosteatosis to nonalcoholic steatohepatitis (NASH). Saturated free fatty acids (SFAs), which are increased significantly in NASH, are directly hepatotoxic which induce hepatocyte lipoapoptosis. Previously, we reported that protein phosphatase 4 (PP4) was a novel regulator of hepatic insulin resistance and lipid metabolism, but its role in hepatic lipoapoptosis remains unexplored. In this study, we found out that PP4 was upregulated in the livers of western diet-fed-induced NASH mice and SFA-treated murine primary hepatocytes and HepG2 cells. In addition, we found for the first time that suppression of PP4 decreased SFA-induced JNK activation and expression of key modulators of hepatocyte lipoapoptosis including p53-upregulated modulator of apoptosis (PUMA) and Bcl-2-interacting mediator (Bim) and reduced hepatocyte lipoapoptosis level as well both in vitro and in vivo. Further study revealed that PP4 induced JNK activation and lipoapoptosis-related protein expression by regulating the RAC1/MLK3 pathway instead of the PERK/CHOP pathway. The effects of palmitate-treated and PP4-induced lipoapoptosis pathway activation were largely abolished by RAC1 inhibition. Moreover, we identified that PP4 interacted with RAC1 and regulated GTPase activity of RAC1. In conclusion, these results demonstrated that PP4 was a novel regulator of hepatocyte lipoapoptosis and mediated hepatocyte lipoapoptosis by regulating the RAC1/MLK3/JNK signaling pathway. Our finding provided new insights into the mechanisms of this process.

## 1. Introduction

The occurrence of large amounts of hepatocytes death is one fundamental difference between benign steatosis and progressive nonalcoholic steatohepatitis (NASH) [1]. Lipotoxicity-induced apoptosis, also referred to as lipoapoptosis, is one of the main modes of hepatocyte death that contributes to NASH progress [2, 3].

Circulating levels of saturated free fatty acids (SFAs) are increased significantly in NASH as compared to hepatosteatosis [4]. SFAs are taken up by hepatocytes in the liver, and excess of SFAs are directly hepatotoxic which induce lipoapoptosis [5]. The activated JNK pathway (mainly JNK 1 and 2 in hepatocytes) is the central pathway of SFA-induced hepatocyte lipoapoptosis [3, 6]. Activated JNK pathway induces the upregulation of proapoptotic Bcl-2 homology3- (BH3-) only proteins (PUMA and Bim), which are key regulators of hepatic lipoapoptosis [7, 8]. These proapoptotic signaling cascades further induce mitochondrial outer membrane permeabilization, the activation of effector caspases, and subsequent lipoapoptosis procession [3, 5]. Two major pathways have been demonstrated to mediate SFA-induced JNK activation, including the ras-related C3 botulinum toxin substrate 1(RAC1)/mixed lineage kinase 3 (MLK3) pathway [5, 9, 10] and endoplasmic reticulum (ER) stress pathway [5, 11–13]. But the molecular mechanisms involved in hepatocyte lipoapoptosis are incompletely understood.

Protein phosphatase 4 (PP4) is an evolutionarily conserved protein serine/threonine phosphatase that belongs to the PP2A family, and mouse PP4 protein shares 100% amino acid sequence identity with human PP4 [14]. It has been shown to participate in multiple cellular processes, including the DNA damage response [15], cell proliferation [16], and multiple signaling pathways regulation [14, 17]. Recently, our group verified the role of PP4 in hepatic insulin resistance and lipid metabolism. PP4 forms a complex with IRS-1 and participates in inflammatory-related hepatic insulin resistance [18]. PP4 promotes hepatic lipogenesis by dephosphorylating ACC-1 [19]. Additionally, PP4 interacted with hnRNPU and involved in proliferation of HepG2 cells [16]. However, the contribution of PP4 to SFA-induced hepatocyte lipoapoptosis has not been explored.

To determine the role of PP4 on hepatocyte lipoapoptosis, the expression level of PP4 in human HepG2 cells, murine primary hepatocytes, and western diet-fed-induced NASH mice was decreased either by PP4 siRNA or by AD-PP4shRNA transfection/injection and increased by AD-PP4 transfection/injection. We demonstrated that PP4 promoted PUMA and Bim upregulation through a RAC1/MLK3/JNK-dependent signaling pathway, which led to hepatocyte lipoapoptosis. Moreover, PP4 interacted with RAC1 and regulated the GTPase activity of RAC1. Therefore, our findings suggested that PP4 was a positive regulator of RAC1/MLK3/JNK pathway and participated in hepatocyte lipoapoptosis.

## 2. Material and Methods

### 2.1. Antibodies

Antibodies against *β*-actin (#4970), Bim (#2933), caspase 3 (#9662), p-PERK (#3179), CHOP (#2895), JNK (#9252), p-JNK (#9251), and MLK-3 (#2817) were purchased from Cell Signaling Technology (Boston, Massachusetts, USA). An anti-p-MLK3 (arb6424) antibody was purchased from Biorbyt (orb6424). Anti-RAC1 (155938), anti-p-RAC1 (203884) and anti-PUMA (ab9643) antibodies were purchased from Abcam (Cambridge, UK). Antibodies against PP4 (sc-6118) and PERK (sc-13073) were purchased from Santa Cruz Biotechnology (Dallas, TX, USA).

### 2.2. Preparation of BSA-Bound Palmitate

Palmitate (P5585) and BSA (A2058) were obtained from Sigma-Aldrich Co. (St Louis, MO, USA). According to a previously described protocol [20], palmitate was dissolved in EtOH at a stock concentration of 250 mM. Palmitate was conjugated with BSA at a 4 : 1 molar ratio before treatment. Specifically, the palmitate stock and 10% BSA was separately incubated in 60°C water bath for 10 min. 480 *μ*l palmitate stock was drop wise added into 20 ml prewarmed 10% BSA to make 6 mM palmitate solution. The palmitate solution should be heated at 60°C water bath for 15 min before experiment. For 600 *μ*M palmitate treatment, DMEM medium containing 1% bovine serum albumin was used for cell culture.

### 2.3. Adenoviral Vectors, Lentiviral Vectors and siRNAs

pAdxsi-GFP-PP4, pAdxsi-GFP-PP4shRNA, and the control adenovirus vector were synthesized by the Chinese National Human Genome Center, as previously described. RAC1 shRNA lentiviral vectors (sc-36351) were purchased from Santa Cruz Biotechnology (Dallas, TX, USA). Two siRNA duplexes (siRNA1: 5′-GGUUACAAGUGGCACUUCATT-3′ and siRNA2: 5′-GGACGAGCAUCUCC AGAAATT-3′) targeting human PP4 were used to deplete PP4 (Genepharm, Shanghai, China). A nonspecific siRNA duplex was used as control. siRNA oligomers were transfected using HiPerFect transfection reagent (Qiagen, Hilden, Germany) according to the manufacturer's instructions.

### 2.4. Cell Culture and Treatment

Mouse primary hepatocytes were isolated as previously described [18]. HepG2 cells purchased from ATCC were cultured in high-glucose DMEM supplemented with 10% fetal bovine serum (HyClone), 100 U/ml penicillin (Gibco), and 0.1 mg/ml streptomycin (Gibco). For palmitate treatment, cells were fasted in serum-free DMEM for 12 h before being treated with 600 *μ*M palmitate conjugated with 1% bovine serum albumin for 48 h. For AD-PP4, AD-PP4shRNA, and RAC1shRNA lentiviral treatment, cells were transfected with adenovirus at a 30 multiplicity of infection (MOI) before treatment with palmitate. Each experiment was carried out at least 3 separate times.

### 2.5. Animals and Treatment

C57BL/6J male mice were purchased from the Peking University Health Science Center (Beijing, China) and housed in individual cages in a temperature-controlled environment with a 12-h light/dark cycle. Six- to eight-week-old C57BL/6J mice were fed a standard chow diet or a western diet (41% kcal from fat, Research Diet mD12079B, USA) for 16 weeks to establish a diet-induced NASH model [21].

Mice were intravenously injected through the tail vein with control adenovirus (control AD), PP4 (AD-PP4), or an inhibitor (AD-PP4shRNA) at a dose of 5 × 10^8^ plaque-forming units (PFU) in 100 *μ*l PBS (0.1 ml/25 g body weight). Mice were sacrificed, and tissues were harvested on day 9 after adenovirus injection. All animal experiments were performed in accordance with recommendations of the National Research Council Guide for Care and Use of Laboratory Animals, and the protocols were approved by the Animal Ethics Committee of the Beijing Hospital (2018BJYYEC-164-01).

### 2.6. Western Blot Analysis

Equal amounts of lysate (15-30 *μ*g) were resolved by SDS-PAGE and transferred to polyvinylidene difluoride membranes (Millipore Corp., Bedford, MA, USA). Membranes were blocked in Tris-buffered saline containing 0.1% Tween 20 (TBST) containing 5% nonfat skim milk at room temperature for 2 h and probed with primary antibodies overnight at 4°C. After five washes in TBST, membranes were incubated with 1 : 5000 horseradish peroxidase-conjugated secondary antibodies in TBST for 1 h (Santa Cruz Biotechnology). Bands were visualized using enhanced chemiluminescence western blotting detection reagents (Amersham Pharmacia Biotech, Inc., Piscataway, NJ, USA).

### 2.7. Histological Analysis of Tissues

Liver samples were fixed in optical cutting temperature (O.C.T.) compound (Tissue-Tek, Japan) and sectioned at a thickness of 8 *μ*m. Slides were stained with oil red O, hematoxylin and eosin (H&E), or TUNEL according to the manufacturer's instructions.

### 2.8. Detection of Apoptotic Cells

For TUNEL staining analysis, the nuclear fragmentation of cryosections and cells was detected by a TUNEL staining kit (Roche). Cells in 10 randomly chosen fields from each dish were counted to semiquantitatively determine the ratio of the apoptotic nuclei. Each data point indicates the results from 1600 to 2000 cells in 3 independent experiments.

For DNA laddering analysis, cells were lysed in lysis buffer (10 mM Tris-Cl, 150 mM NaCl, 10 mM EDTA, 0.4% SDS, and 100 *μ*g/ml protease K), incubated at 37°C for 12 h, then extracted with phenol/CHCl3/isoamyl alcohol, and followed by CHCl3/isoamyl alcohol. DNA fragmentation was detected by loading 10 *μ*g of total DNA onto 2.5% agarose gel.

### 2.9. GTPase Activation Assay

RAC1 activation was analyzed using a GTPase activation assay kit purchased from Upstate Biotechnology according to the manufacturer's instructions. Briefly, cell lysates were incubated with 10 *μ*g of glutathione S-transferase- (GST-) agarose beads coupled to the GST-Pak-1-binding domain (PBD). Samples were subjected to SDS-PAGE and immunoblotting. Membranes were probed with a mouse monoclonal antibody against RAC1 (Abcam) followed by western blot analysis. The amount of PBD-bound RAC1 was normalized to the amount of protein in cell lysates.

### 2.10. Immunoprecipitation

Immunoprecipitation was performed as previously described [16]. In brief, HepG2 cells were lysed in lysis buffer. PP4 was immunoprecipitated with an anti-PP4 antibody (Sc-6118). RAC1 was immunoprecipitated with an anti-RAC1 antibody (ab203884). The immunoprecipitates were washed three times with washing buffer containing 50 mM HEPES (pH 7.4), 0.1% Triton X-100, and 500 mM NaCl.

### 2.11. Statistical Analysis

All data are presented as the mean ± SD. The statistical significance of the differences between various treatments was determined by Student's *t*-test or one-way ANOVA (Tukey-Kramer honestly significant difference). A *p* value less than 0.05 was considered significant.

## 3. Results

### 3.1. PP4 Expression Level Was Upregulated by Palmitate-Treated Hepatocytes

**Figure 1:**
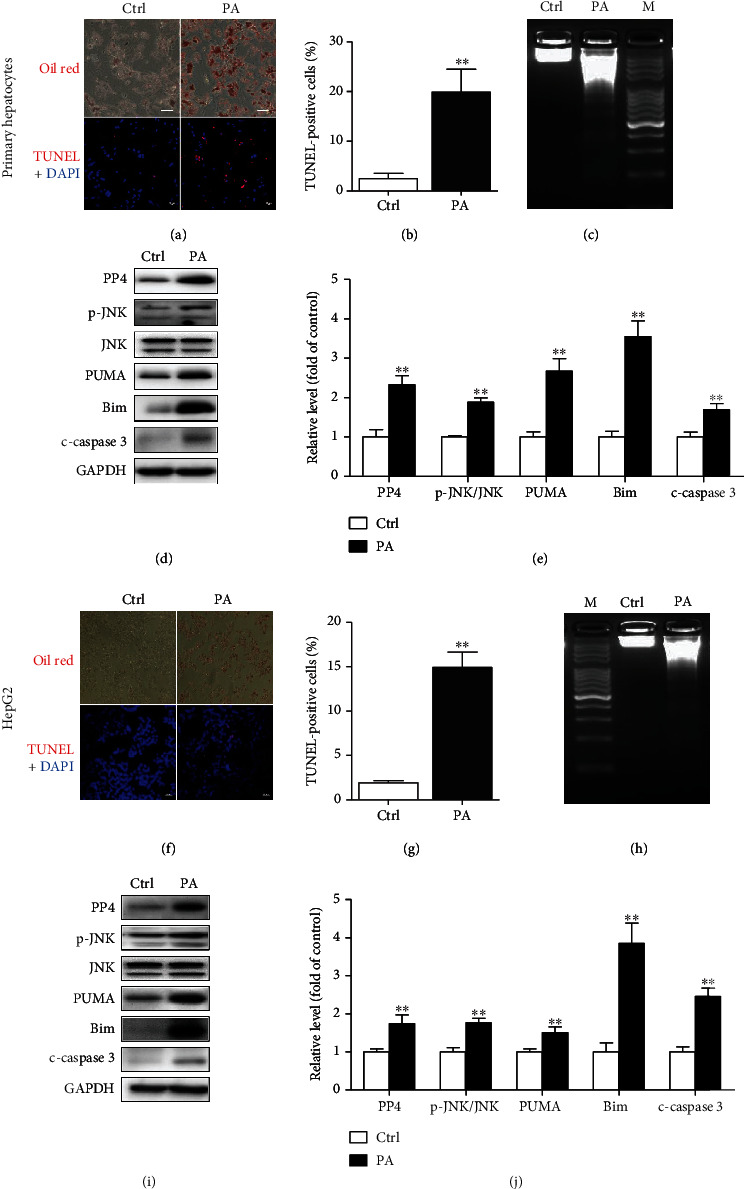
PP4 expression level was upregulated in palmitate-treated hepatocytes. After treatment with 600 *μ*M palmitate for 48 h, oil red O staining showed lipid accumulation and TUNEL staining showed apoptotic cells in murine primary hepatocytes (a) and HepG2 cells (f) (scale bar: 20 *μ*M). TUNEL-positive dots were measured by the ImageJ software in murine primary hepatocytes (b) and HepG2 cells (g) (*n* = 10 images per group). Apoptosis of murine primary hepatocytes (c) and HepG2 cells (h) was detected by DNA laddering in palmitate-treated cells. Protein levels of PP4, PUMA, Bim, c-caspase 3, and JNK activation were measured by western blot in murine primary hepatocytes (d) and HepG2 cells (i). Relative protein levels were quantified by densitometry using the ImageJ software and normalized to GAPDH in murine primary hepatocytes (e) and HepG2 cells (j). Data are expressed as means ± SD (*n* = 5), and statistical significance for protein levels was determined by Student's *t*-test (^∗∗^*p* < 0.01).

Hepatocyte lipoapoptosis can be induced in vitro by incubating hepatocytes with SFAs. First, we examined PP4 expression in the hepatic lipoapoptosis cell model. Mouse primary hepatocytes treated with palmitate 600 *μ*M for 24 h. Oil red staining showed significant lipid accumulation (Figure 1(a)). Apoptosis level elevated as indicated by TUNEL staining (Figures 1(a) and 1(b)) and DNA ladder (Figure 1(c)). Along with JNK activation and the upregulated expression of lipoapoptosis-related proteins such as PUMA, Bim, and c-caspase 3, the expression of PP4 significantly increased following palmitate treatment (Figures 1(d) and 1(e)). Moreover, we did the phenotype analysis in HepG2 cells treated with palmitate, and similar results were obtained from HepG2 cells (Figures 1(f)–1(j)). These data demonstrated that palmitate could induce hepatic lipoapoptosis and upregulate the expression of PP4.

### 3.2. PP4 Regulated Palmitate-Induced Lipoapoptosis in Hepatocytes

**Figure 2:**
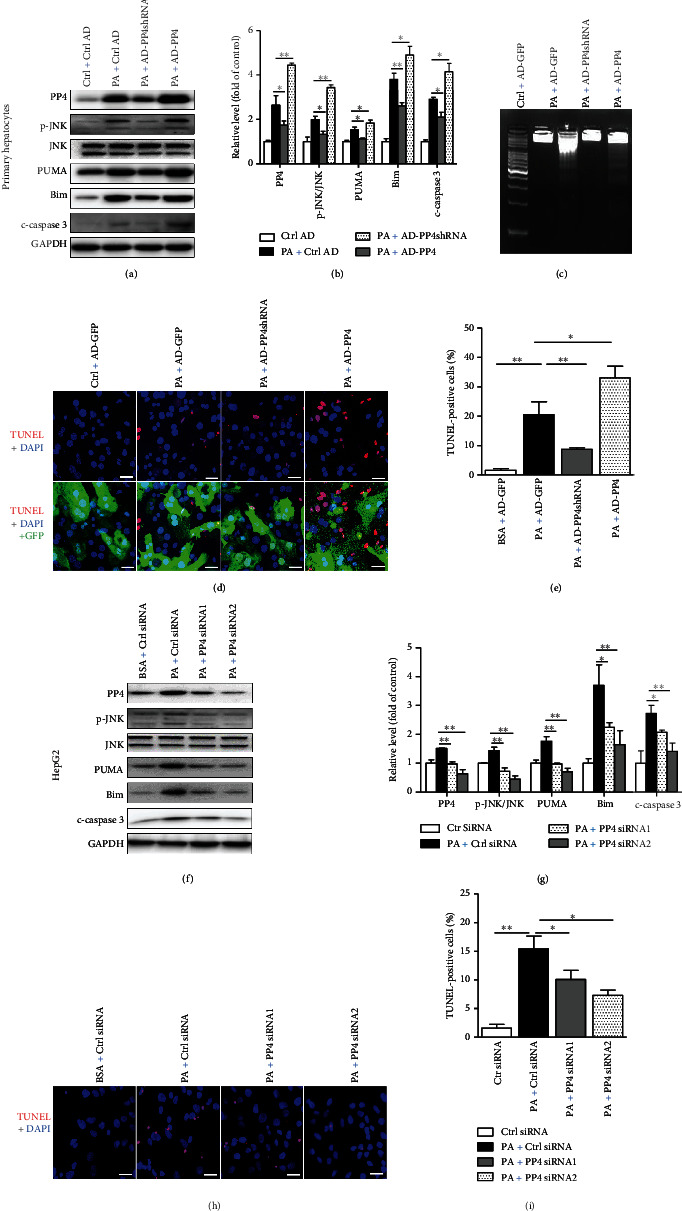
PP4 regulated palmitate-induced lipoapoptosis in hepatocytes. Primary hepatocytes were pretreated with control AD, AD-PP4shRNA, and AD-PP4 for 24 h followed by palmitate treatment. HepG2 was transfected with control siRNA and 2 pairs of PP4 siRNAs for 24 h followed by palmitate treatment. Protein expressions of PP4, PUMA, Bim, c-caspases, and phosphorylation levels of JNK were measured by western blot analysis in murine primary hepatocytes (a) and HepG2 cells (f). Relative protein levels were quantified by densitometry and normalized by GAPDH (b, g). Apoptotic cells were detected by DNA laddering in murine primary hepatocytes (c). TUNEL staining showed apoptotic cells in murine primary hepatocytes (d) and HepG2 cells (h). TUNEL-positive dots were analyzed by the ImageJ software (e, i).

To investigate whether PP4 is involved in hepatic lipoapoptosis, we inhibited PP4 expression with the recombinant adenoviral vectors expressing PP4shRNA (AD-PP4shRNA) and upregulated PP4 expression by recombinant adenoviral vectors expressing PP4 (AD-PP4) in mouse primary hepatocytes. Silencing PP4 by AD-PP4shRNA transfection reduced palmitate-induced JNK activation and PUMA, Bim and c-caspase 3 expression level (Figures 2(a), 2(b)). Meanwhile, DNA ladder analysis (Figure 2(c)) and TUNEL staining (Figures 2(d) and 2(e)) indicated that PP4 inhibition decreased hepatic lipoapoptosis level. Moreover, upregulation of PP4 promoted palmitate-induced lipoapoptosis signaling pathway and lipoapoptosis level (Figures 2(a)–2(e)), which further confirmed the role of PP4 in hepatocyte lipoapoptosis. In addition, we did the phenotype analysis in HepG2 cells treated with palmitate, and similar results were obtained from siRNA-mediated PP4 inhibition in HepG2 cells (Figures 2(f)–2(i)).

Together, these data suggested that knockdown of PP4 could restore palmitate-induced hepatic lipoapoptosis, and overexpression of PP4 promoted palmitate-induced lipoapoptosis.

### 3.3. PP4 Regulated Lipoapoptosis in the Liver of Western Diet-Fed Mice

**Figure 3:**
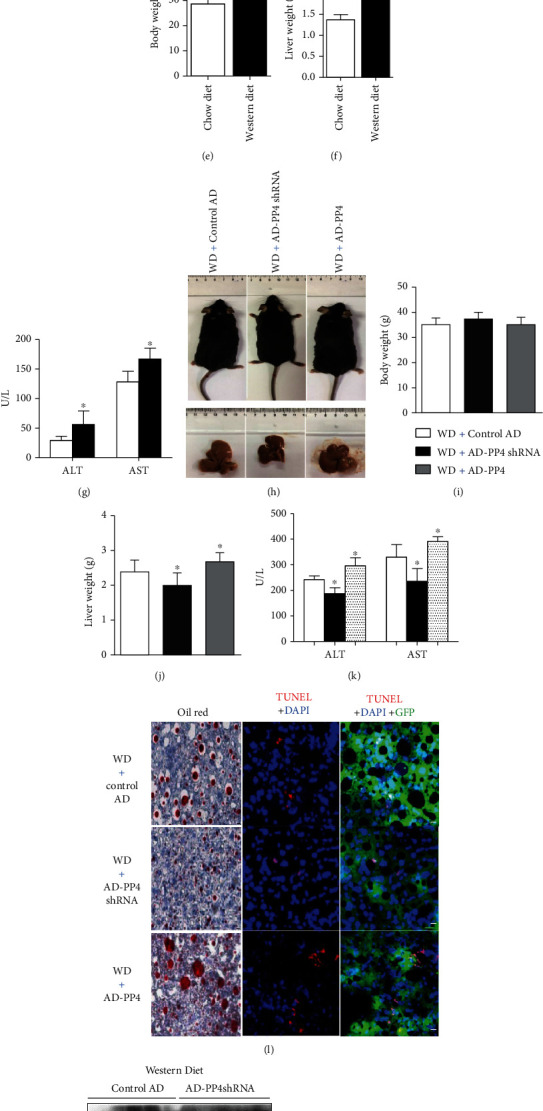
PP4 regulated lipoapoptosis in the liver of western diet-fed mice. Six- to eight-week-old male C57BL/6J mice fed with western diet for 16 weeks were injected with control AD, AD-PP4shRNA, or AD-PP4 (*n* = 5 for each group) through the tail vein and sacrificed 10 days postinjection. Body weight and liver weight in western diet-fed mice (a, e, f) and western diet-fed mice injected with control AD, AD-PP4shRNA, or AD-PP4 (h–j). Oil red O staining, TUNEL staining, and/or H&E staining of frozen liver sections in western diet-fed mice (scale bar: 50 *μ*M) (b) and western diet-fed mice injected with control AD, AD-PP4shRNA, or AD-PP4 (l) (scale bar: 20 *μ*M). Serum ALT and AST levels were measured in western diet-fed mice (g) and western diet-fed mice injected with control AD, AD-PP4shRNA, or AD-PP4 (k). Western blots of hepatic PP4, PUMA, Bim, c-caspase 3, and phosphorylation levels of JNK in western diet-fed mice (c), western diet-fed mice injected with AD-PP4shRNA (m), and AD-PP4 (o). Relative protein levels were quantified by densitometry and normalized by GAPDH (d, n, p). Data are expressed as means ± SD, and statistical significance for protein levels was determined by one-way ANOVA (Tukey-Kramer honestly significant difference), (^∗^*p* < 0.05, ^∗∗^*p* < 0.01).

To determine whether PP4 is involved in hepatic lipoapoptosis in vivo, we analyzed its expression in the mouse liver after western diet treatment (Figure 3(a)). Feeding with western diet for 16 weeks induced significant increase of body weight and liver weight (Figures 3(e) and 3(f)) in C57BL/6J mice. In addition, serum alanine aminotransferase (ALT) and aspartate aminotransferase (AST) levels were elevated in mice fed with western diet compared with control mice (Figure 3(g)). Oil red O and TUNEL staining revealed a significant increase of lipid deposition and hepatic apoptosis rate in the livers of western diet-fed mice (Figure 3(b)). Compared with control mice, western diet-fed mice exhibited elevated JNK phosphorylation and lipoapoptosis-related protein expressions in the livers, including PUMA, Bim, and cleaved-caspase 3 (Figures 3(c) and 3(d)). In addition, western diet-fed mice showed a significant increase in hepatic PP4 expression (Figures 3(c) and 3(d)).

To determine whether PP4 is involved in hepatic lipoapoptosis, we suppressed PP4 expression in the liver of western diet-fed mice with AD-PP4shRNA by administering tail vein injections. Ten days after administration, the expression level of hepatic PP4 was reduced to ~40% by AD-PP4shRNA compared with control adenovirus vectors (Control AD) (Figures 3(m) and 3(n)). Compared with Control AD, AD-PP4shRNA treatment did not alter mouse body weight but decrease liver weight and serum levels of ALT and AST (Figures 3(h)–3(k)). Moreover, the 60% reduction in PP4 expression decreases hepatic lipid deposition and hepatic lipoapoptosis level (Figure 3(l)). In addition, this effect was associated with decreased expression levels of PUMA, Bim, c-caspase 3 protein, and phosphorylation level of JNK in the liver (Figures 3(m) and 3(n)). These results suggest that inhibition of PP4 expression in the liver may prevent hepatic lipoapoptosis in western diet-fed mice. Additionally, we upregulated PP4 expression in the liver of western diet-fed mice with AD-PP4. Our data showed that the upregulation of PP4 did not alter body weight but elevated liver weight and aggravated liver injury (Figures 3(h)–3(k)) by promoting western diet-induced lipid deposition, lipoapoptosis (Figure 3(l)), and lipoapoptosis-related signaling pathway activation (Figures 3(o) and 3(p)), which is consistent with the phenotype of PP4 inhibition.

Together, PP4 regulated lipoapoptosis in the liver of western diet-fed mice.

### 3.4. PP4 Regulated JNK Activation through a RAC1/MLK3 Pathway in Hepatocytes

**Figure 4:**
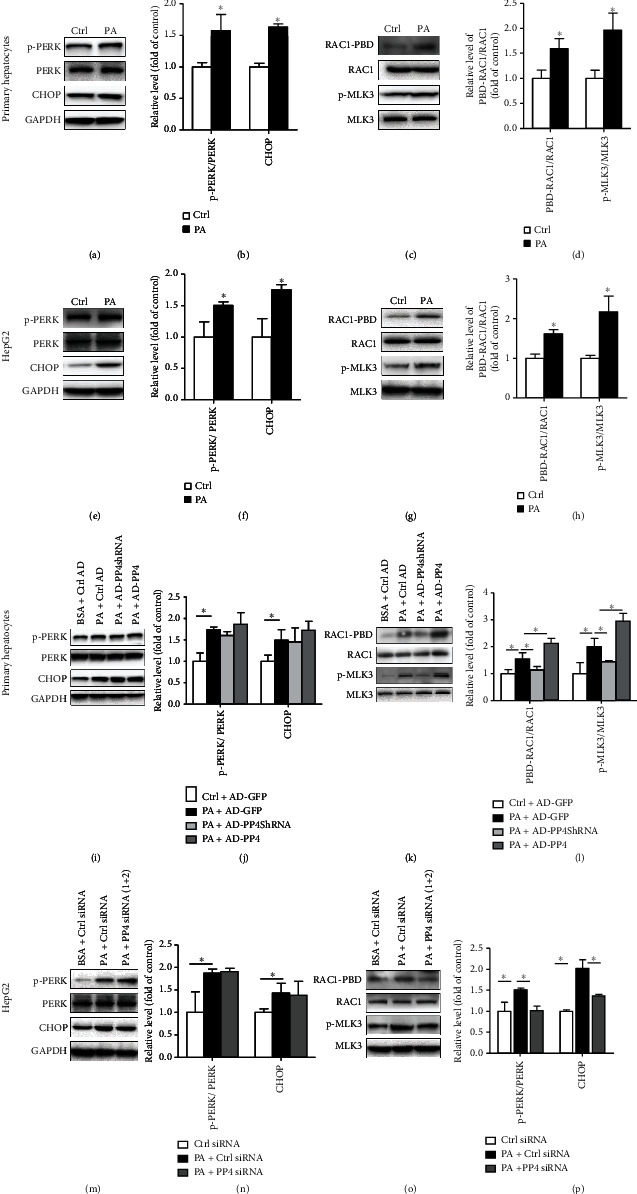
PP4 regulated JNK activation through a RAC1/MLK3 pathway in hepatocytes. Endoplasmic reticulum (ER) stress and RAC1/MLK3 signaling pathways were measured in palmitate-treated primary hepatocytes and HepG2 cells, PP4 inhibited and PP4 upregulated primary hepatocytes and HepG2 cells. Protein expressions of CHOP and phosphorylation levels of PERK and MLK3 were measured by western blot analysis. RAC1 activation was determined by binding to GST-PAK PBD and subsequent immunoblot analysis in palmitate-treated murine primary hepatocytes (a, c), HepG2 cells (e, g), AD-PP4shRNA and AD-PP4 pretreated murine primary hepatocytes (i, k), and PP4 siRNA-transfected HepG2 cells (m, o). Relative protein levels were quantified by densitometry and normalized (b, d, f, h, j, l, n, p). Data are expressed as means ± SD, and statistical significance for protein levels was determined by Student's *t*-test or one-way ANOVA (^∗^*p* < 0.05).

Endoplasmic reticulum (ER) stress and RAC1/MLK3 signaling are 2 important pathways involved in palmitate-induced JNK activation and lipoapoptosis. So, we analyzed the effect of PP4 on these pathways. Consistent with previous studies, palmitate activated ER stress as indicated by elevated phosphorylation level of PERK and upregulation of CHOP both in mouse primary hepatocytes (Figures 4(a) and 4(b)) and HepG2 cells (Figures 4(e) and 4(f)). Simultaneously, palmitate led to elevated RAC1 activity as indicated by elevated combination ability of Pak-binding domain (PBD). Also, MLK3 was phosphorylated rapidly following palmitate treatment both in primary hepatocytes (Figures 4(c) and 4(d)) and HepG2 cells (Figures 4(g) and 4(h)).

Then, we analyzed the effect of PP4 on these pathways. As shown in Figures 4(i)–4(l), inhibition of PP4 decreased RAC1 activation and the MLK3phosphorylation (Figures 4(k) and 4(l)) but did not affect the activation of PERK and the expression and CHOP (Figures 4(i) and 4(j)) in mouse primary hepatocytes. Also, overexpression of PP4 enhanced the phosphorylation of MLK3 (Figures 4(k) and 4(l)) but did not affect the PERK/CHOP signaling (Figures 4(i) and 4(j)). Similar results were obtained in palmitate-treated HepG2 cells. MLK3 instead of PERK/CHOP signaling significantly activated following PP4 siRNA transfection (Figures 4(m)–4(p)).

### 3.5. PP4 Regulated JNK Activation through a RAC1/MLK3 Pathway In Vivo

**Figure 5:**
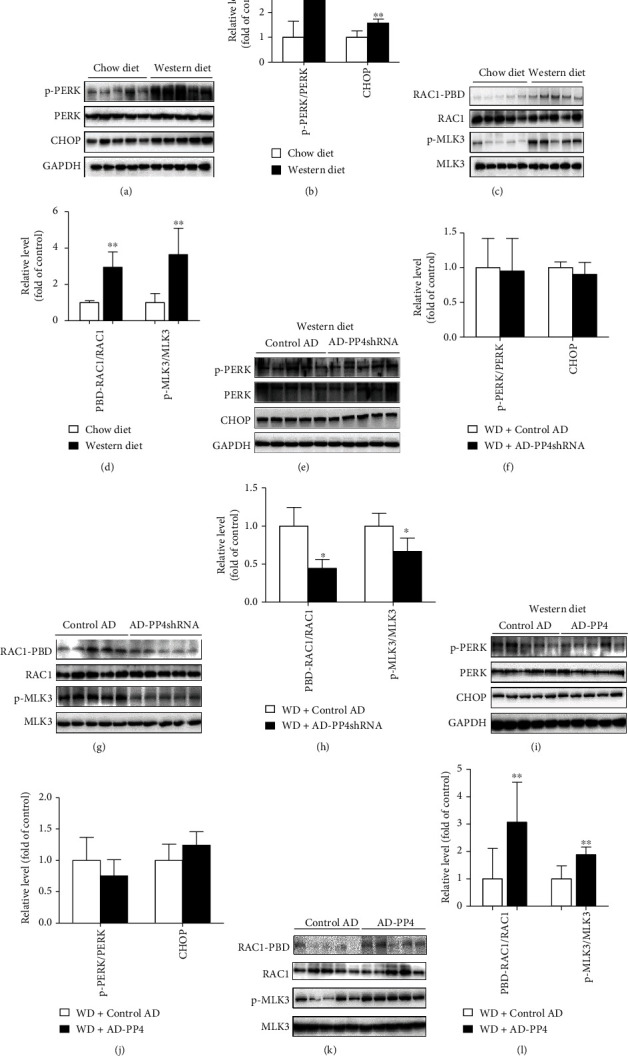
PP4 regulated JNK activation through a RAC1/MLK3 pathway in vivo. Endoplasmic reticulum (ER) stress and RAC1/MLK3 signaling pathways were measured in western diet-fed mice and mice injected with control AD, AD-PP4shRNA, or AD-PP4 through the tail vein. Protein expressions of CHOP and phosphorylation levels of PERK and MLK3 were measured by western blot analysis. RAC1 activation was determined by binding to GST-PAK PBD and subsequent immunoblot analysis in western diet-fed mice (a, c) and AD-PP4shRNA- (e, g) and AD-PP4-injected (i, k) mice. Relative protein levels were quantified by densitometry and normalized (b, d, f, h, j, l). Data are expressed as means ± SD, and statistical significance for protein levels was determined by Student's *t*-test (^∗^*p* < 0.05, ^∗∗^*p* < 0.01).

Consistent with previous in vitro study, activation of the PERK/CHOP (Figures 5(a) and 5(b)) and RAC1/MLK3 signaling (Figures 5(c) and 5(d)) were also observed in the liver of western diet-fed mice. The suppression of PP4 expression by AD-PP4shRNA did not induce significant change of the PERK/CHOP pathway (Figures 5(e) and 5(f)) but had significant effect on RAC1/MLK3 signaling activation (Figures 5(g) and 5(h)). Also, upregulation of PP4 expression by AD-PP4 did not induce significant change of the PERK/CHOP pathway (Figures 5(i) and 5(j)) but had significant effect on RAC1/MLK3 signaling activation (Figures 5(k) and 5(l)).

Taken together, these results demonstrated that PP4 participated in hepatic JNK activation by activating RAC1/MLK3 signaling instead of PERK/CHOP pathway.

### 3.6. RAC1 Mediates PA-Induced MLK3/JNK Activation and Lipoapoptosis

**Figure 6:**
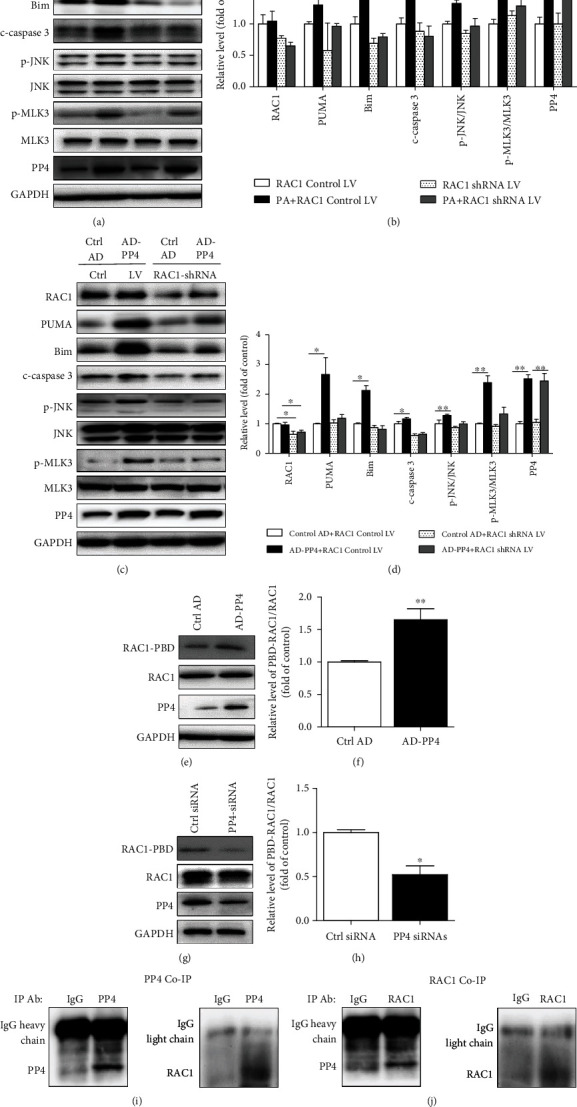
PP4 regulated lipoapoptosis through regulating the activity of RAC1. HepG2 cells were pretreated with control LV and RAC1 shRNA LV for 24 h followed by palmitate treatment or AD-PP4 transfection. The levels of RAC1, PUMA, Bim, PP4, and c-caspase 3 and phosphorylation levels of JNK and MLK3 were measured by western blot analysis in palmitate treatment cells (a) and AD-PP4-transfected cells (c). Relative protein levels were quantified by densitometry and normalized (b, d). RAC1 activity was determined by binding to GST-PAK PBD and subsequent immunoblot analysis in AD-PP4- (e, f) and PP4 siRNA- (g, h) transfected cells. HepG2 cells were transfected with control AD or AD-PP4 for 24 h. The interaction between PP4 and RAC1 was confirmed using coimmunoprecipitation with an anti-PP4 antibody (i) and RAC1 antibody (j). Data are expressed as means ± SD, and statistical significance for protein levels was determined by Student's *t*-test or one-way ANOVA (^∗^*p* < 0.05, ^∗∗^*p* < 0.01).

To verify the role of RAC1 in hepatic lipoapoptosis, the RAC1 expression was suppressed by RAC1-shRNA lentivirus (LV) transfection in HepG2. As shown in Figures 6(a) and 6(b), in palmitate-treated HepG2 cells, RAC1 suppression led to decreased MLK3/JNK activation and PUMA, Bim, and c-caspase 3 expression levels induced by palmitate. Moreover, RAC1 inhibition did not induce significant change in PP4 expression.

### 3.7. PP4 Regulated Lipoapoptosis through Regulating the Activity of RAC1

Previous studies showed that PP4 was a positive regulator of Rho GTPases, including RAC1 in HEK293 cells by regulating their GTP-binding activity [22]. So, we speculated that PP4 may induce hepatic lipoapoptosis through regulating RAC1 activation. Next, we assessed the role of RAC1 in PP4-mediated lipoapoptosis. We found that in RAC1-silenced cells, PP4-induced MLK3/JNK activation was abolished by RAC1 inhibition (Figures 6(c) and 6(d)). More importantly, the knockdown of RAC1 reduced PP4 upregulation-induced PUMA, Bim, and c-caspase 3 levels. Taken together, these data suggested that RAC1 was a key mediator in PP4 induced lipoapoptosis.

Then, we analyzed the effect of PP4 on RAC1 activity. We found that PP4 upregulation by AD-PP4 transfection led to RAC1 activation (Figures 6(e) and 6(f)), and PP4 suppression by siRNA transfection decreased the levels of PBD-RAC1 (Figures 6(g) and 6(h)).

We addressed whether PP4 was able to regulate RAC1 directly. Coimmunoprecipitation revealed that RAC1 was immunoprecipitated with PP4 by an anti-PP4 antibody (Figure 6(i)) and vice versa (Figure 6(j)). Taken together, we identified RAC1 as a novel interaction protein with PP4 and PP4-regulated lipoapoptosis through regulating the activity of RAC1.

## 4. Discussion

In this study, we provided new insights into the mechanisms of hepatocyte lipoapoptosis and described a positive function of PP4 in this model. We had demonstrated for the first time that (1) PP4 was a positive regulator of hepatocyte lipoapoptosis both in vitro and in vivo, (2) PP4 promoted JNK-mediated lipoapoptosis by activating the RAC1/MLK3 instead of PERK/CHOP signaling pathway, and (3) PP4 interacted with RAC1 and regulated RAC1 GTPase activity. Each of these results is discussed in greater detail below.

Lipoapoptosis induced by excess toxic lipids is one of the main modes of hepatocyte death that contributes to NASH progress [2]. Accumulating evidence have shown that the SFAs can induce hepatic lipoapoptosis both in vivo and in vitro. In hepatocytes, the activation of JNK signaling pathways is central regulator of hepatic lipoapoptosis [6, 7]. Two types of BH3-only proteins, PUMA and Bim, were reported to be key sensitizers of hepatocytes to lipoapoptosis, which further triggers mitochondrial outer membrane permeabilization [23], the activation of the executioner caspases 3 and 7, and cell death [3, 5]. But the molecular mechanisms involved in hepatocyte lipoapoptosis are incompletely understood.

PP4 is a novel regulator of hepatic glucose and lipid metabolism. Previously, our group showed that PP4 formed a complex with IRS-1 and participated in inflammatory-related insulin resistance [18]. Additionally, PP4 promoted hepatic lipogenesis by dephosphorylating ACC-1 [19]. However, the contribution of PP4 to SFA-induced hepatocyte lipoapoptosis has not been explored. In this study, we found that along with elevated levels of hepatocyte apoptosis and the activation of lipoapoptosis signaling, the expression of PP4 significantly increased in the liver of western diet-fed mice, palmitate-treated HepG2 cells, and murine primary hepatocytes, suggesting that PP4 may be involved in hepatocyte lipoapoptosis.

In order to verify the important role of PP4 on lipoapoptosis, we assessed the regulatory effect of PP4 on these regulators both in vitro and in vivo. In murine primary hepatocytes and HepG2 cells, the inhibition of hepatic PP4 expression reduced hepatic lipoapoptosis and was accompanied by decreased expression levels of lipoapoptosis-related molecules. Additionally, the PP4 upregulation increased lipoapoptosis and activated related molecules. On the other hand, to examine the role of PP4 in hepatic lipoapoptosis in vivo, we suppressed PP4 expression in the liver of western diet-fed mice with AD-PP4shRNA and upregulated PP4 expression with AD-PP4 by administering tail vein injections. The knockdown of PP4 reversed western diet-induced lipoapoptosis signaling. It is noteworthy that liver weight and lipoapoptosis were ameliorated in the AD-PP4 shRNA-injected mice. Also, PP4 upregulation promoted western diet-induced lipid deposition, lipoapoptosis level, and lipoapoptosis-related signaling pathway activation. These data confirmed that PP4 contributed to hepatic lipoapoptosis.

How is PP4 involved in hepatic lipoapoptosis? In the liver, both RAC1/MLK3 [5, 9, 10] and ER stress [11–13, 24] signaling contribute to SFA-induced JNK activation. In accordance with previous studies, we found that the RAC1/MLK3 signaling pathway and ER stress pathway were simultaneously activated when hepatocytes underwent lipoapoptosis. While inhibition of PP4 had significant effect on RAC1/MLK3 activation, it did not affect the activation of ER stress signaling both in vitro and in vivo. Similarly, the upregulation of PP4 expression also enhanced hepatocyte lipoapoptosis by activating the MLK3/JNK signaling pathway instead of the PERK/CHOP pathway both in vivo and in vitro. These data strongly suggested PP4 likely regulated hepatocyte lipoapoptosis via the MLK3/JNK signaling pathway.

What is the mechanism underlying PP4 involvement in the palmitate-induced RAC1/MLK3 pathway activation? In palmitate-treated hepatocytes, RAC1, a key type of GTP-binding protein, has been reported to be an important component of a SFA-stimulated signaling pathway that regulates MLK3-dependent activation of JNK in hepatocytes [9, 25]. RAC1 is activated by palmitate treatment and participates in MLK3/JNK activation. The combination of RAC1 and the CRIB motif of MLK3 releases the self-inhibition of MLK3, followed by the activation of the MLK3/JNK signaling pathway [9, 26]. In accordance with a previous study, palmitate stimulation increased the GTPase activity of RAC1 in hepatocytes, while silencing RAC1 expression in HepG2 cells significantly inhibited the activation of the MLK3/JNK signaling pathway induced by palmitate. Our data confirmed that palmitate-induced JNK activation and lipoapoptosis were through a RAC1-dependent pathway. Moreover, PP4 has been reported to be a positive regulator of Rho GTPases, including RAC1, in HEK293 cells by regulating their GTP-binding activity [22]. So, we speculated that PP4 may induce hepatic lipoapoptosis through regulating RAC1 activation. As we expected, PP4 also regulates the GTPase activity of RAC1 in hepatocytes. The upregulation of PP4 enhances the GTPase activity of RAC1, while the suppression of PP4 significantly reduces its GTPase activity. Moreover, RAC1 inhibition significantly inhibited PP4-induced MLK3/JNK pathway activation. In addition, we found PP4 was able to bind RAC1 directly by coimmunoprecipitation. Our data indicated that PP4 regulated lipoapoptosis through regulating the activity of RAC1.

However, how PP4-regulated RAC1 activity needed further verification? For many years, RAC1 activity is believed to be regulated only by the activation family guanine nucleotide exchange factors (GEFs), inhibition family GTPase-activating proteins (GAPs), and guanine nucleotide disassociation inhibitors (GDIs) [26]. Recent studies show that reversible phosphorylation is also involved in the regulation of RAC1 activity. Ser71 (64yrplsyp73) is a conserved Akt site on RAC1 that can be phosphorylated by Akt. The phosphorylation of Ser71 by AKT inhibits the GTP-binding activity of RAC1, which further inhibits the activation of downstream signals, including the JNK signaling pathway [27]. Another study shows that thr108 (106pntp109) is an ERK phosphorylation site of RAC1. It can be phosphorylated by ERK and induces the translocation of RAC1 from the cytoplasm to the nucleus, which prevents the transmission of extracellular signals to the cell [28]. In addition, there are more than 20 candidate phosphorylation sites in RAC1 by PhosphoSite analysis, but whether these sites participate in the regulation of RAC1 activity needs further verification. For many years, due to the lack of specific phosphatase motifs, research on protein dephosphorylation has been relatively lagging. To date, the phosphatase that dephosphorylates the ser71, thr108, and other phosphorylation sites of RAC1 were still unknown, and whether these sites were regulated by PP4 needed further verification.

**Figure 7:**
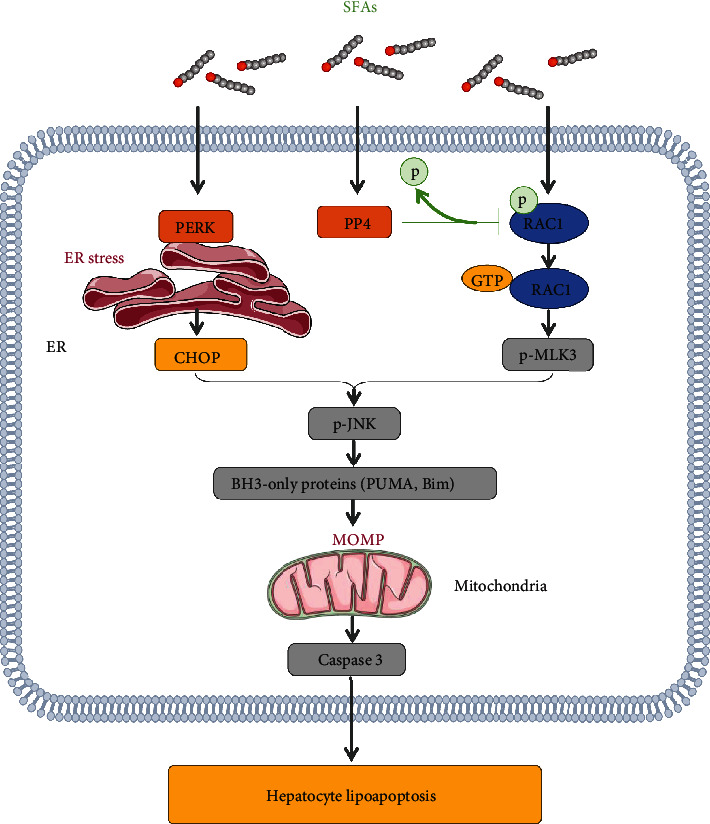
Molecular mechanism by which PP4 participates in hepatocyte lipoapoptosis. Stimulation of PP4 by SFAs causes enhanced GTPase activity of RAC1 possibly by dephosphorylating RAC1, which subsequently activate MLK3/JNK pathway, which led to elevated expression of Bcl-2 proapoptotic family members and promoted hepatocyte lipoapoptosis.

In conclusion, increased expression of hepatic PP4 contributed to lipoapoptosis by enhancing the GTPase activity of RAC1 and activating the MLK3/JNK pathway, which led to elevated expression of Bcl-2 proapoptotic family members and promoted hepatocyte lipoapoptosis (Figure 7). Overall, we revealed the significant role of PP4 in hepatocyte lipoapoptosis, which may provide benefit in developing novel therapeutic approaches for NASH treatment in the future.

## Data Availability

All data needed to evaluate the conclusions in the paper are present in the paper and/or the Supplementary Materials. Additional data related to this paper may be requested from the authors.
